# Genome-wide association mapping of nutritional traits for designing superior chickpea varieties

**DOI:** 10.3389/fpls.2022.843911

**Published:** 2022-08-23

**Authors:** Manish Roorkiwal, Aditi Bhandari, Rutwik Barmukh, Prasad Bajaj, Vinod Kumar Valluri, Annapurna Chitikineni, Sarita Pandey, Bharadwaj Chellapilla, Kadambot H. M. Siddique, Rajeev K. Varshney

**Affiliations:** ^1^Center of Excellence in Genomics & Systems Biology, International Crops Research Institute for the Semi-Arid Tropics (ICRISAT), Hyderabad, India; ^2^The UWA Institute of Agriculture, The University of Western Australia, Perth, WA, Australia; ^3^Khalifa Center for Genetic Engineering and Biotechnology (KCGEB), United Arab Emirates University, Al Ain, Abu Dhabi, United Arab Emirates; ^4^ICAR- Indian Agricultural Research Institute (IARI), New Delhi, India; ^5^State Agricultural Biotechnology Centre, Centre for Crop and Food Innovation, Murdoch University, Murdoch, WA, Australia

**Keywords:** biofortification, micronutrient, malnutrition, trait-mapping, genomics, GWAS

## Abstract

Micronutrient malnutrition is a serious concern in many parts of the world; therefore, enhancing crop nutrient content is an important challenge. Chickpea (*Cicer arietinum* L.), a major food legume crop worldwide, is a vital source of protein and minerals in the vegetarian diet. This study evaluated a diverse set of 258 chickpea germplasm accessions for 12 key nutritional traits. A significant variation was observed for several nutritional traits, including crude protein (16.56–24.64/100 g), β-Carotene (0.003–0.104 mg/100 g), calcium (60.69–176.55 mg/100 g), and folate (0.413–6.537 mg/kg). These data, combined with the available whole-genome sequencing data for 318,644 SNPs, were used in genome-wide association studies comprising single-locus and multi-locus models. We also explored the effect of varying the minor allele frequency (MAF) levels and heterozygosity. We identified 62 significant marker-trait associations (MTAs) explaining up to 28.63% of the phenotypic variance (PV), of which nine were localized within genes regulating G protein-coupled receptor signaling pathway, proteasome assembly, intracellular signal transduction, and oxidation–reduction process, among others. The significant effect MTAs were located primarily on Ca1, Ca3, Ca4, and Ca6. Importantly, varying the level of heterozygosity was found to significantly affect the detection of associations contributing to traits of interest. We further identified seven promising accessions (ICC10399, ICC1392, ICC1710, ICC2263, ICC1431, ICC4182, and ICC16915) with superior agronomic performance and high nutritional content as potential donors for developing nutrient-rich, high-yielding chickpea varieties. Validation of the significant MTAs with higher PV could identify factors controlling the nutrient acquisition and facilitate the design of biofortified chickpeas for the future.

## Introduction

Malnutrition, also known as hidden hunger, is a global nutritional problem mainly affecting women, infants, children, and adolescents. One or more forms of malnutrition abound in every country, with multifaceted impacts on humans through increased morbidity, disability, stunted mental growth, reduced productivity and economic growth, and serious and lasting social and medical bearings (FAO, IFAD, UNICEF, WFP, and WHO, 2017). Micronutrient malnutrition affects more than 2 billion people globally (Ritchie and Roser, 2020). Approximately every second pregnant woman and 40% of pre-school children in developing countries are anemic. According to the World Health Organization (WHO) projections, neonatal deaths will increase from 46% in 2016 to 52% in 2030 (FAO, IFAD, UNICEF, WFP, and WHO, 2017). In 2020, WHO estimated that about 149 million children under 5 years suffer from stunting, while 38.9 million are overweight or obese. The Global Hidden Hunger Index, one of several metrics indicating the severity of micronutrient malnutrition, is defined as “alarmingly high” across several countries in South Asia (SA) and sub-Saharan Africa (SSA). Modern breeding strategies and agronomic practices during the Green Revolution have significantly increased major cereal crop productivity (Bouis and Welch, 2010), but a simultaneous increase in micronutrient malnutrition in many nations, calls for a re-evaluation of agricultural efforts to provide a healthy mix of sufficient calories and essential nutrients. A sustainable solution to malnutrition should link agriculture to nutrition and health (Jones and Ejeta, 2016).

Among food crops, legumes serve as an inexpensive and key source of protein (20–25%), minerals [iron (Fe), magnesium (Mg), potassium (K), phosphorus (P), and zinc (Zn)], vitamins (B1, B2, B3, B6, and B9), and omega-3 fatty acids, compared to cereals (White and Broadley, 2009). Numerous studies have documented the significance of legumes to complement starches derived from cereals and root crops, and facilitate nutrient absorption (Jukanti et al., 2012; Mudryj et al., 2014; Sánchez-Chino et al., 2015; Foyer et al., 2016). In regions such as SA and SSA, legumes have a substantial socio-economic impact on driving food and nutritional security. To address the challenges related to micronutrient malnutrition and the non-availability of nutritious food, selective breeding approaches that tap into the genetic variation of nutritional traits in the legume germplasm pool will ensure nutritional balance even in adverse ecologies. This potential of conventional breeding to increase micronutrient density by exploiting and selecting genetic variation from breeding material has been established in different studies (Graham and Welch, 1996; Graham et al., 1999, 2001). Micronutrient density traits are stable across environments. Significant examples include identifying orange-flesh sweet potato lines with high levels of β-Carotene (>200 μg/g), beans with improved agronomic traits, seed type, and 50–70% more iron, and yellow cassava, orange maize, iron pearl millet, zinc rice, and zinc wheat bread using conventional breeding strategies by HarvestPlus (HarvestPlus Annual Report, 2015). Transgenic approaches are useful in this context and, in some cases, can be advantageous over conventional breeding. However, regulatory and political restrictions for using transgenic approaches limit their applicability, best exemplified by Golden Rice. However, there is compelling evidence, based on micronutrient deficiency rates, that biofortification is feasible for nutritional improvement without compromising agronomic traits, in addition to the current objectives of developing climate-resilient varieties with biotic and abiotic stress tolerance (Garcia-Casal et al., 2017; Rehman et al., 2019).

Chickpea (*Cicer arietinum* L.) is one of the largest cultivated food legumes globally and a major source of protein in the vegetarian diet (Jukanti et al., 2012). Chickpea protein is the best among all legume proteins, as it has good *in vitro* protein digestibility (Sánchez-Vioque et al., 1999; Yust et al., 2003). It is also a highly valued source of other nutrients, such as carbohydrates, minerals, vitamins, fats, fibers, lipids, and oils. Annual global chickpea production is about 14.25 million metric tons cultivated on 13.72 million hectares, with India accounting for about 70% of global production (FAOSTAT, 2019). However, many SA and SSA countries consume cereal-based diets deprived of nutrients and bioavailability.

The dawn of the genomics era in the 21st century has significantly increased the understanding of genomics research in bacterial, plant, and animal species. For example, crop improvement efforts in chickpea have greatly benefited from the rapid development of high-throughput genotyping technologies generating molecular markers to determine the origin and diversity of populations (Nayak et al., 2010; Thudi et al., 2011; Roorkiwal et al., 2013), elucidating gene expression of complex agronomic traits (Varshney et al., 2013a; Mannur et al., 2019; Rezaei et al., 2019; Bharadwaj et al., 2020; Roorkiwal et al., 2020; Barmukh et al., 2022), and genome sequencing and characterization (Jain et al., 2013; Varshney et al., 2013b, 2019). These significant developments have irreversibly influenced plant breeding, redefining it as “genomics-assisted breeding” (GAB; Varshney et al., 2005). Shifting the plant breeding paradigm from “breeding by design” to “genome-wide approaches,” genome-wide association studies (GWAS) have become a popular approach to accelerate breeding, as selections are based on marker-trait associations (MTAs) as a response to the combined effect of all favorable alleles. Breeding programs for varietal development were initially challenged with a low transfer of well-characterized genes/QTL, as the genomic regions of interest were identified in biparental populations. Alternatively, association mapping in diversity panels has accelerated the identification of genomic regions associated with agronomic traits by detecting ancestral recombination events that caused the non-random association of alleles at different loci across the genome. Association mapping, in turn, enables a higher mapping resolution than the biparental linkage analysis (Zhu et al., 2008).

With the availability of this vast wealth of genomic resources, there is much scope to study the nutritional traits in chickpea, identify MTAs/QTL using molecular markers, and integrate them in GAB programs (Rehman et al., 2019; Roorkiwal et al., 2021). GWAS has gained tremendous momentum in legumes, with numerous studies reporting markers linked to Fe and Zn concentration in lentils (*Lens culinaris* Medik; Khazaei et al., 2017), seed copper (Cu), P and K concentrations in chickpea (Ozkuru et al., 2018), Fe chlorosis in soybean (Mamidi et al., 2014; Assefa et al., 2020), and Fe bioavailability in cooked dry beans (*Phaseolus vulgaris* L.; Katuuramu et al., 2018).

In view of the above, the present study aimed to identify MTAs for 12 nutritional traits in the chickpea reference set (Upadhyaya et al., 2008), using three different algorithms. These include one single-locus [mixed linear model (MLM)] and two multi-locus [multi-locus mixed model (MLMM) and Bayesian-information and linkage-disequilibrium iteratively nested keyway (BLINK)] algorithms. Furthermore, we explored the effect of markers by varying the minor allele frequency levels and heterozygosity parameters. The latter was found to significantly affect the detection of associations contributing to the traits of interest. Finally, we identified some potential micronutrient-rich accessions that can be used as donors in chickpea breeding programs.

## Materials and methods

### Germplasm accessions and evaluation

The chickpea genotype-based reference set comprises of 300 diverse accessions (267 landraces, 13 advanced breeding lines and cultivars, seven accessions of wild *Cicer*, and 13 of unknown biological statuses), as described in Upadhyaya et al. (2008). Of these, 280 were evaluated in this study for 12 key nutritional traits, namely β-Carotene, calcium (Ca), crude protein, folate/vitamin B9 (Fo), iron (Fe), magnesium (Mg), manganese (Mn), phytic acid, vitamin B1 (Vit B1), vitamin B2 (Vit B2), vitamin B6 (Vit B6), and zinc (Zn), at the National Collateral Management Services Ltd. (NCML), Vishakapatnam, India. Seeds of the accessions were acquired from the ICRISAT gene bank. The analyte concentration measurement procedures for unprecedented trace impurity detection and sensitive quantitation of nutrient elements, using different spectroscopy methods and combustion analyses, are explained below.

### Elemental analysis for Ca, Fe, Mg, Mn, and Zn

About 0.5 g of homogenized seed sample was weighed into a digestion tube and digested with suprapure nitric acid using a microwave digester (Make: Anton Parr, Model: Multiwave Go). The digestate was filtered using Whatman® filter paper no. 42 and made up to a volume of 10 ml using ultrapure water in a calibrated volumetric flask.

Analyte concentrations were determined using inductively coupled plasma–optical emission spectroscopy (ICP-OES; Make: Perkin Elmer, Model: 7300DV). Calibration was performed using a blank and five matrix-matched standards. The calibration curve was fitted using linear regression with a minimum acceptable correlation coefficient of 0.995. Ca, Fe, Mg, Mn, and Zn were analyzed in radial mode using standard method (Method 984.27). Unknown samples were quantified using Winlab 32 software.

### β-Carotene analysis

About 1 g of homogenized seed sample was placed into a 50 ml centrifuge tube with 6 ml of 1% ascorbic acid (prepared in ethanol) and then vortexed for 2 min. Next, 0.5 ml of 80% KOH was added to the mixture, vortexed, and heated at 80°C for 10 min. Subsequently, 3 ml of cool water and extraction solvent (n-hexane) was added, vortexed for 2 min, and centrifuged for 5 min at 3,500 rpm.

The supernatant layer was collected for the Turbo evaporator in the Ria vial. The analysis was repeated by adding 3 ml n-hexane, then vortexing and centrifuging for 5 min at 3,500 rpm. The supernatant layer was separated into the previously transferred vial, and the sample was dried in a Turbo evaporator. The sample was reconstituted in 1 ml methanol, sonicated, and centrifuged for 5 min at 3,500 rpm. Finally, the samples were transferred into instrument vials and injected into the ultra-performance liquid chromatography- photodiode array detector (UPLC-PDA) for analyzing the β-Carotene content (Make: Waters Acquity UPLC H-Class) following standard method as described earlier (Sundaresan, 2002).

### B-complex analysis

About 1 g of homogenized seed sample was placed into a 50 ml centrifuge tube with 10 ml HPLC grade water. The sample was vortexed for 2 min and then centrifuged for 5 min at 3,500 rpm. The supernatant layer (1 ml) was collected in a 15 ml centrifuge tube containing 9 ml water. The sample was vortexed and filtered through 0.45 μm filter paper.

The filtered sample was collected in a Ria vial (500 μl), added 500 μl of reagent water. The sample was mixed thoroughly and transferred into instrument vials. The prepared sample was injected into the liquid chromatography- tandem mass spectrometry detector (LC–MS/MS; Make: Waters XEVO TQ-S Micro) for B-complex analysis using standard protocol (Martin et al., 2016).

### Phytic acid analysis

About 1.0 g of finely powdered seed sample was weighed into a centrifuge tube. An aqueous solution of 5% trichloroacetic acid (TCA; 25 ml) was added to the sample, vortexed for 2 min, and placed in a water bath at 60°C. The mixture was cooled and vortexed again for 2 min and centrifuged at 3,000 rpm for 5 min. The precipitate was washed twice with 5% TCA and centrifuged at 3,000 rpm for 5 min. The supernatants were pooled, and the volume was made up to 50 ml with 5% TCA. An aliquot of 20 ml was placed into a centrifuge tube before adding 5 ml of 0.25% FeCl3. The tube was heated in a water bath at 95°C for 45 min. The contents were cooled, and volume made up to 100 ml with distilled water, prepared against reagent blank. The available ferric ion was determined by reaction with potassium thiocyanate (KSCN), which developed a blood-red color. A 1 ml aliquot was transferred to a 50 ml volumetric flask, and 10 ml of 29% KSCN was added. The solution was calibrated with distilled water and immediately read for color at 480 nm in a spectrophotometer (Thermo Evolution 201). A reagent blank was run with each set of samples, and a standard curve was plotted into series (0.1–5.0 mg/L). A total of 10 ml of 29% KSCN was added to the mixture, and the samples were immediately analyzed at 480 nm. Phytate content was calculated from the iron concentration by assuming a constant Fe:P molecular ratio of 4:6 in the precipitate using standard method (Wheeler and Ferrel, 1971).

### Crude protein analysis

About 0.5 g of finely powdered seed sample was weighed into a Pelican digestion tube before adding 0.7 g mercury oxide, 15 g powdered anhydrous sodium sulfate, and 25 ml H2SO4. The test tubes were placed on a heater in the digestion unit and gently heated until foaming ceased and then boiling vigorously until the solution became clear. It took about 2 h for the sample to turn pale green or light blue. After complete digestion, the digestion unit was switched off, and the insert rack with digested samples was lifted and placed on a rack stand with an exhaust manifold system. This facilitated fast cooling and removed scares acid fumes. In the distillation step, 0.1 N HCl was added from the reservoir’s hose to the receiver conical flask, and 40% NaOH was added to the located sample digestion tube. The ammonia and steam coming from the sample were consolidated in the distillation process. The ammonia was passed through a glass water condenser.

The ammonia was collected in the receiver solution, removed, and taken to a manual burette for titration with 0.1 N NaOH solution, using methyl red as an indicator. The equipment used was a Pelican Nitrogen Analyzer for combustion based analysis for nitrogen (CLASSIC-DX) using Indian standard method (IS: 7219-1973).

### Analysis of phenotypic data

An initial diagnostic analysis was run on each trait using the “influence” option based on Mahalanobis distance using the R-MVN package (R. v.1.2.5001) to detect potential outliers among the individual data points. Based on the biological status of the accessions, eight accessions categorized as “others” were filtered out. A further quality check of the phenotypic data filtered out 14 accessions to avoid spurious associations. The working set of 258 accessions (G1) was used for further analyses. The “corrplot” package in R (R. v.1.2.5001) was used to estimate Pearson’s correlation among the measured traits, while the “Factoextra” R package was used to undertake the principal component analysis (PCA) for the filtered data. The frequency distribution plots were generated using the “ggplot2” package in the R environment.

### Genotypic data

The genotypic data for the 280 accessions were obtained from the database of Centre of Excellence in Genomics and Systems Biology, ICRISAT (https://cegsb.icrisat.org/openaccessdata; 27). The raw genotypic data extracted from the database contained 1,115,262 SNPs distributed on eight pseudomolecules, Ca1–Ca8. The filtering for missing data (≤20%) and minor allele frequency (MAF) ≥2% for Ca1–Ca8 using vcftools led to the first working set of 318,644 SNPs (referred to as 318 K) and an additional filter for the rate of heterozygosity (Ho) ≤0.5% led to a working set of 73,968 SNPs (referred as 74 K) in the second working set. To further explore the effect of MAF and Ho levels, we generated matrices with MAF ≥5% and Ho ≤5% (Supplementary Table 1).

### Genetic structure

Genetic diversity among the accessions of the working set was studied with the 318 K marker matrix using the neighbor-joining (NJ) clustering method in TASSEL 5 (Bradbury et al., 2007) and visualized using FigTree v1.4.3 (Rambaut, 2016). The population structure was assessed using ADMIXTURE v.1.3.0 (Alexander et al., 2009), with the results visualized using the R/pophelper (Francis, 2017) package. A series of models for K values ranging from 2 to 8 were run with 5-fold cross-validation to prime the main algorithm (QuasiNewton) for convergence acceleration. Accuracy and precision were ensured by performing 20 runs for each value of *K*, and the *K* value determined the optimal number of clusters with the least cross-validation error.

### Association analysis

We performed GWAS with MLM, MLMM, and BLINK models using the R/GAPIT 3.0 package and visualization of circular Manhattan and Quantile-Quantile (Q-Q) plots using the rMVP package (0.99.17; https://github.com/xiaolei-lab/rMVP). The spurious associations in GWAS were corrected using “Bonferroni Correction” (5% level of significance). Further, R2 values were generated using the lm function in R. The percent phenotypic variance (PV) explained by all significant SNPs detected was output from all models used. The PV explained by each significant SNP was calculated as the squared correlation between the phenotype and genotype of the SNP.

### Identification of candidate genes

The genes involving significant SNP markers were aligned against the NCBI non-redundant (nr) protein database using BLASTX to obtain functional annotations. Gene Ontology (GO) and Kyoto Encyclopedia of Genes and Genomes (KEGG) pathway identification were conducted on these sequences in the KEGG pathways in-built in BLAST2GO. The SNPEff-4.3T open source program was used for variant annotation and prediction of significant SNP effects.

### Selection of accessions as potential donors

Promising accessions were selected based on favorable alleles for nutritional traits and yield advantage, observed in phenotypic screening at different environments, as described in Thudi et al. (2014). The premise was to identify a set of accessions that can be incorporated in breeding programs for enhancing micronutrient density without adversely affecting other agronomic traits screened in different environments. Thudi et al. (2014) analyzed the chickpea reference set for 114 agronomic and physiological traits at four locations (Kenya, Ethiopia, and Patancheru and Kanpur in India) over five seasons (spanning 2000–09) and two ecologies (rainfed and irrigated). We analyzed the nutritional traits dataset generated in the present study and datasets from Thudi et al. (2014) for agronomic traits to identify accessions as potential donors for breeding high-yielding varieties with enhanced micronutrient content.

We used two approaches to identify promising accessions from the set of 258 accessions, for superior nutritional and agronomic performance in different environments. In the first approach, accessions with trait values higher than the population median were identified for seed yield (SY; referred as G2) from different environments and the 11 nutritional traits, excluding phytic acid (referred to as G3). The common set of accessions between G2 and G3 were then analyzed for the presence of favorable alleles for superior agronomic performance and micronutrient content. In the second approach, the nutritional data for 12 traits and two agronomic traits, SY and 100-seed weight (100SW from Thudi et al., 2014), were used to identify superior accessions based on trait correlations and hierarchical clustering. A favorable combination of traits was established- higher trait values for SY, 100SW, β-Carotene, Ca, crude protein, Fo, Fe, Mg, Mn, phytic acid, Vit B1, Vit B2, Vit B6, and Zn, and a minimal content of phytic acid. Based on the presence of favorable combination of at least two or more traits, accessions were identified from each cluster for use as potential donors in breeding programs to enhance the genetic potential of chickpea.

## Results

### Distribution and correlation among nutrition traits

**Figure 1:**
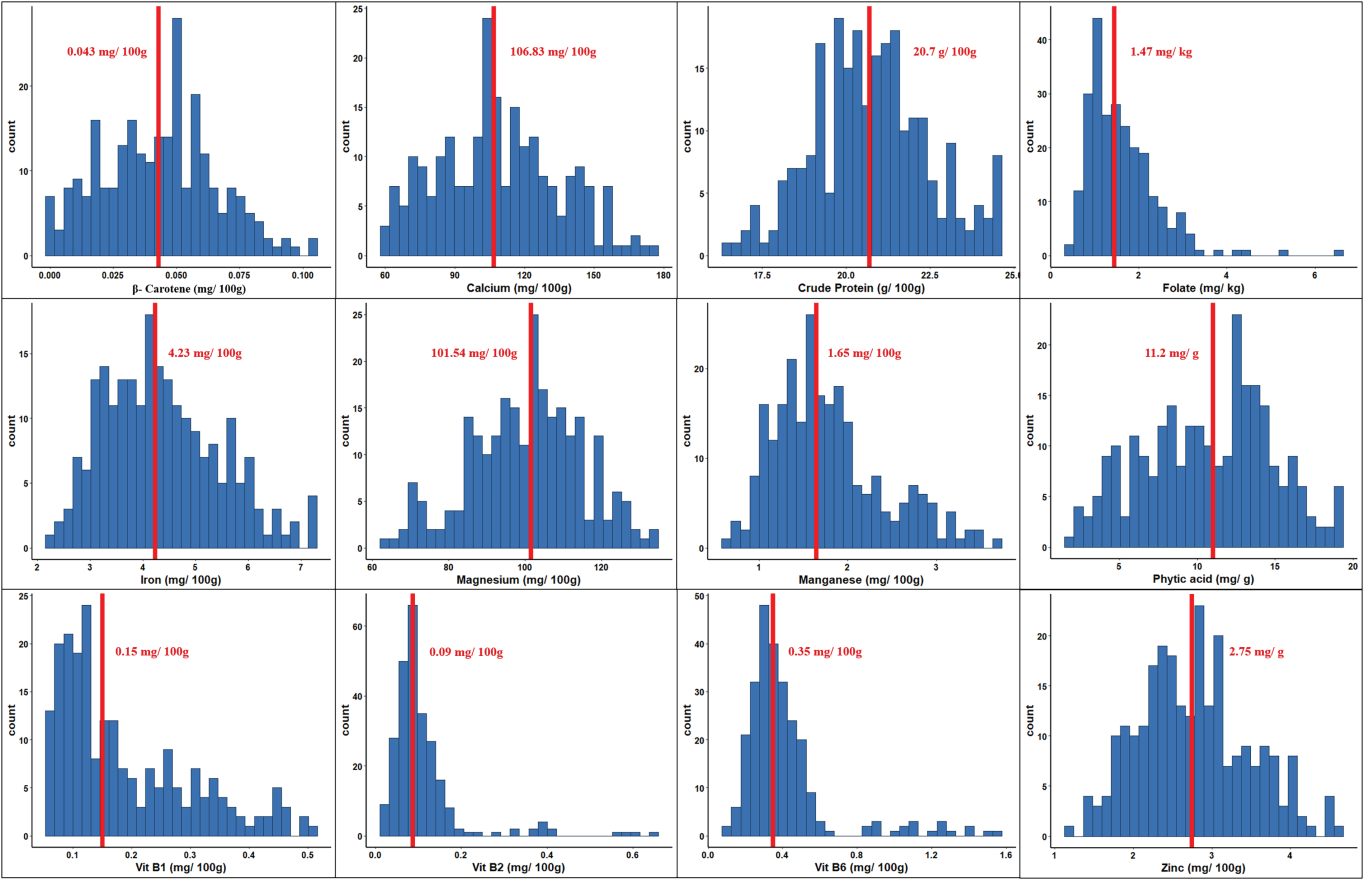
Phenotypic variation for 12 nutritional traits assayed within the chickpea reference set. Within each histogram plot, bold dashed line represents the median. The range and median for each trait are specified in the respective grid.

**Figure 2:**
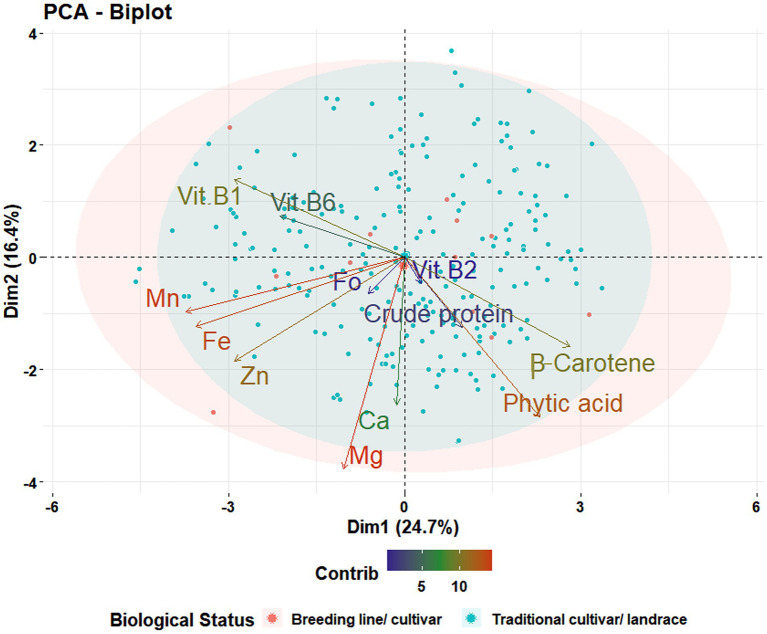
Principal component analysis for 12 nutritional traits. Projection of 258 accessions in the reference set on the first plane of principal component analysis using phenotypic data for 12 traits [β-Carotene, calcium (Ca), crude protein, folate (Fo), iron (Fe), magnesium (Mg), manganese (Mn), phytic acid, vitamin B1 (Vit B1), vitamin B2 (Vit B2), vitamin B6 (Vit B6), and zinc (Zn)]. The first two components, PC1 and PC2, explain 41.1% of the variance between genotypes.

The range and population median for 12 phenotypic variables (β-Carotene, Ca, crude protein, Fo, Fe, Mg, Mn, Zn, phytic acid, and Vit B1, B2, and B6) in 258 accessions from the chickpea reference set are presented in Figure 1. Most traits exhibit a symmetric distribution, except for Fo and Vit B1, B2, and B6, which are skewed. Table 1 shows the broad range of variation in the 12 nutritional traits in the reference set, compared to the data available on the USDA Food Composition Database.^1^ This includes β-Carotene: 0.003–0.104 mg/100 g (mean 0.044), Ca: 60.69–176.55 mg/100 g (108.69), crude protein: 16.56–24.64 g/100 g (20.74), Fo: 0.41–6.54 mg/kg (1.61), Fe: 2.26–7.25 mg/100 g (4.36), Mg: 64.08–134.57 mg/100 g (100.35), Mn: 0.67–3.73 mg/100 g (1.78), phytic acid: 2.07–19.38 mg/g (10.68), Zn: 1.15–4.59 mg/100 g (2.76), Vit B1: 0.055–0.502 mg/100 g (0.189), Vit B2: 0.011–0.638 mg/100 g (0.111), and Vit B3: 0.116–1.57 mg/100 g (0.411). The PCA of the nutrition traits showed that the first two axes explained 41.1% of the total phenotypic variance (Figure 2). The accessions did not cluster based on their biological status but had varying degrees of relatedness. While Mn and Mg contributed the most to the phenotypic variance, crude protein, Fo, and Vit B2 accounted for the least. Fo, Fe, Mg, Mn, and Zn were inversely related to the concentrations of other analyzed traits.

**Table 1:** Descriptive statistics for the key nutritional traits in the chickpea reference set.

Trait	Range	Mean	Standard deviation
β-Carotene (mg/100 g)	0.003–0.104	0.044	0.022
Calcium (mg/100 g)	60.693–176.550	108.686	25.995
Crude protein (g/100 g)	16.560–24.640	20.738	1.721
Folate (mg/kg)	0.413–6.537	1.609	0.798
Iron (mg/100 g)	2.260–7.248	4.359	1.083
Magnesium (mg/100 g)	64.075–134.566	100.345	14.389
Manganese (mg/100 g)	0.672–3.728	1.782	0.618
Phytic acid (mg/g)	2.070–19.380	10.675	4.095
Vitamin B1 (mg/100 g)	0.055–0.502	0.189	0.113
Vitamin B2 (mg/100 g)	0.011–0.638	0.111	0.091
Vitamin B6 (mg/100 g)	0.116–1.570	0.411	0.243
Zinc (mg/100 g)	1.149–4.585	2.762	0.696

**Figure 3:**
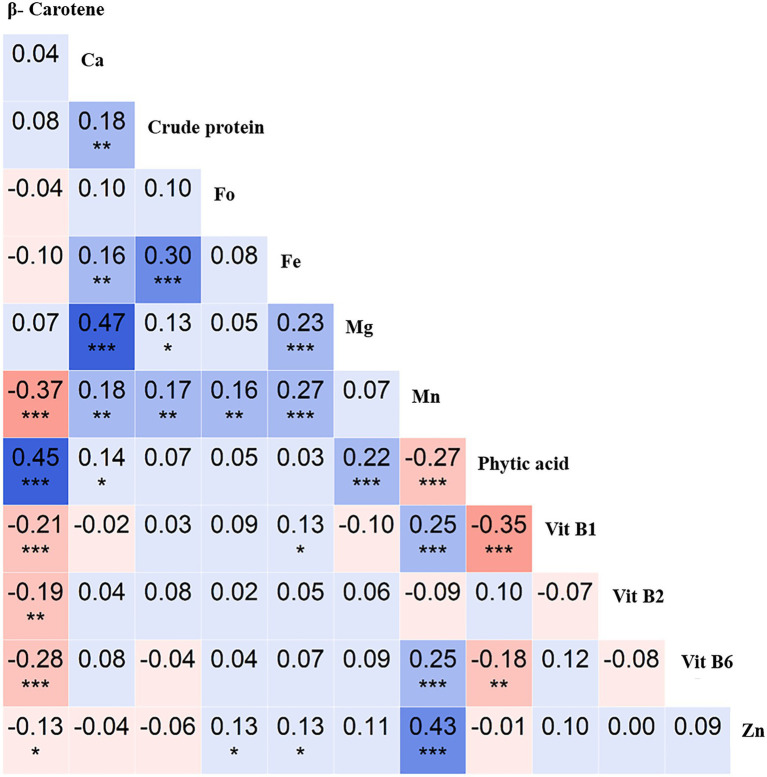
Correlation analysis of 12 nutritional traits evaluated using the chickpea reference set. Pearson’s r-values showing correlations between 12 traits [β-Carotene, calcium (Ca), crude protein, folate (Fo), iron (Fe), magnesium (Mg), manganese (Mn), phytic acid, vitamin B1 (Vit B1), vitamin B2 (Vit B2), vitamin B6 (Vit B6), and zinc (Zn)]. Blue indicates positive correlations, and red indicates negative correlations among traits; color intensity depicts correlation strength. *significant at < 0.05 level, **significant at <0.01 level, *** significant at < 0.001 level, blank for non-significant.

The correlation analysis revealed an interesting trend for the key nutritional traits. For instance, crude protein positively correlated with Ca (*r* = 0.3, *p* < 0.001), as did Ca with Mg (*r* = 0.47, *p* < 0.001), Mn with Zn (*r* = 0.43, *p* < 0.001), and β-Carotene with phytic acid (*r* = 0.45, *p* < 0.001). In contrast, β-Carotene negatively correlated with Mn (*r* = −0.37, *p* < 0.001), as did phytic acid with Vit B1 (*r* = −0.35, *p* < 0.001; Figure 3). These insights will be useful in targeting nutrient biofortification in breeding programs.

### Genotypic characteristics of the population

The MAF and Ho density and distribution of the working set of 318 K loci are summarized in Supplementary Table 2. The Ho distribution varied among Ca1–Ca8, with an average of 0.72%, with more heterozygous calls identified mainly on Ca2, Ca3, Ca4, Ca7, and Ca8. Uneven distribution of markers along the genome was characterized by an average density of 644 markers per Mb. Genomic regions with high marker density were observed on Ca1 and Ca4, with an average magnitude of 766 and 1,364 markers per Mb and high-density regions between 1–8 Mb on Ca1 and 26–46 Mb on Ca4. The distribution of markers along the eight linkage groups is depicted in Supplementary Figure 1.

**Figure 4:**
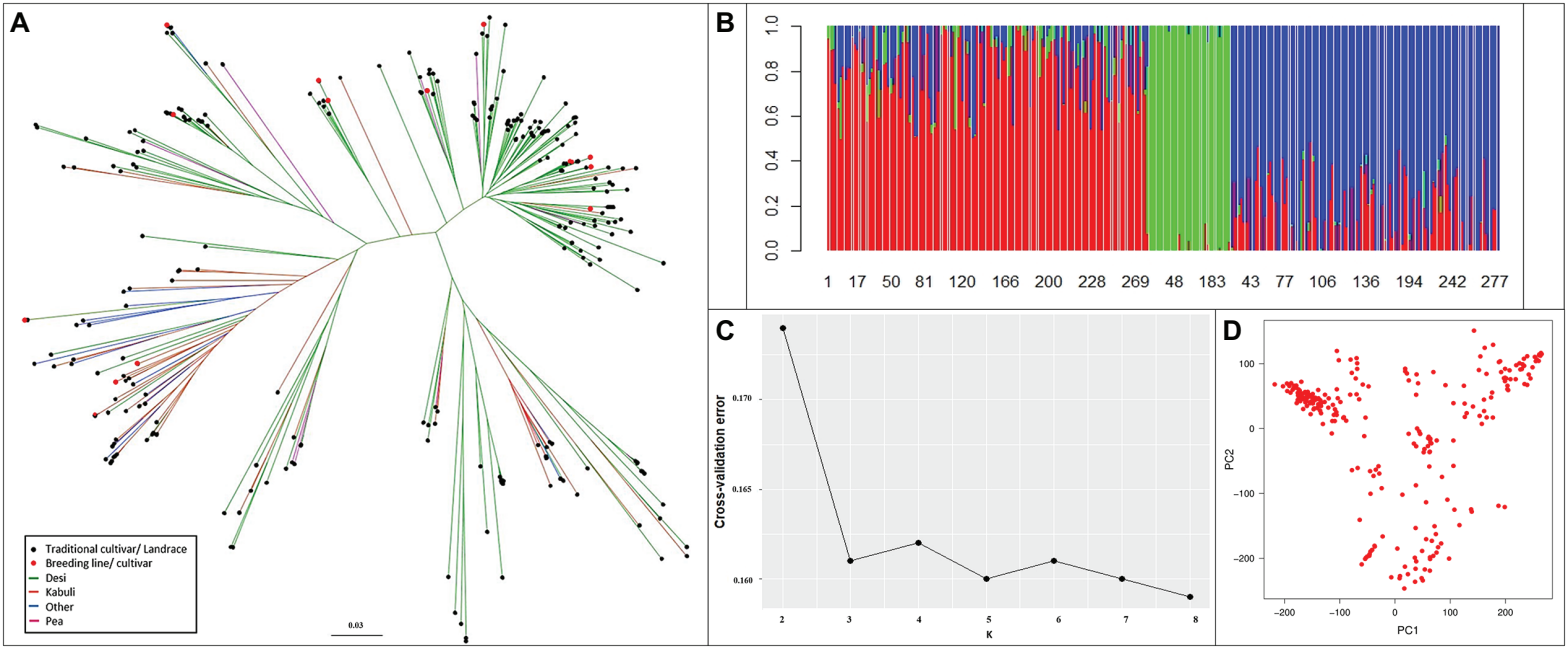
Genetic relatedness and population structure of the reference set. **(A)** Diversity using unweighted neighbor-joining tree method. **(B)** Ancestry proportions from ADMIXTURE analysis (*k* = 3), optimal with lowest cross-validation error. Each colored vertical line indicates the proportion of ancestral population (k) for each accession. The numbers on X-axis represent the reference set accessions. **(C)** Cross-validation error for *k* = 2–8 from ADMIXTURE analysis. **(D)** Variation depicted as PCA plot. Clustering pattern independent of biological status and seed type.

Phylogenetic diversity illustrated by the unweighted neighbor-joining tree (Figure 4A) revealed that the reference set clustered independently to biological status and seed type. The clustering pattern was validated in the population structure analysis; with an optimal K value with the least cross-validation error of 3 (Figures 4B,C). The PCA output of R/GAPIT (Figure 4D) illustrated an indistinct yet broad grouping into three clusters. Therefore, population stratification was accounted for using three principal components, included as covariates in models for association analyses.

### GWAS reveals genomic regions associated with nutritional traits

Three models (MLM, MLMM, and BLINK) detected 11, 18, and 44 MTAs, respectively, for the 12 traits analyzed. Different thresholds of MAF and Ho significantly corrected for spurious associations in MTA detection using a stringent Ho threshold. The number of MTAs detected using genotypic parameters of MAF ≥ 0.02, MAF ≥ 0.02 + Ho ≤ 0.5, and MAF ≥ 0.02 + Ho ≤ 0.05 were 73, 69, and 63, respectively, for all three models combined. The corresponding MTAs detected with genotypic parameters of MAF ≥ 0.05, MAF ≥ 0.05 + Ho ≤ 0.5, and MAF ≥ 0.05 + Ho ≤ 0.05 were 41, 14, and 5, respectively, across all three models.

**Figure 5:**
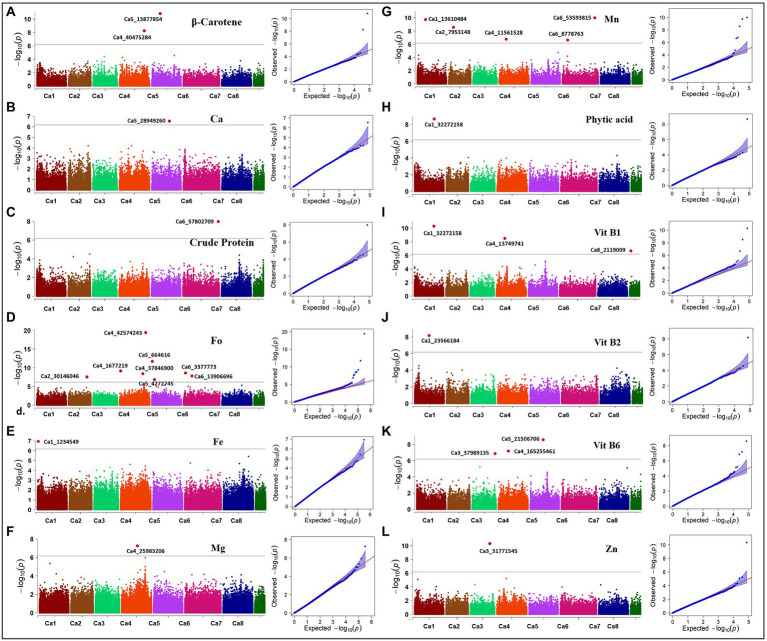
Manhattan plots and Q-Q plots showing association in the diverse reference set for 12 nutritional traits. Manhattan and QQ-plots depicted for **(A)** β-Carotene, **(B)** calcium (Ca), **(C)** crude protein, **(D)** folate (Fo), **(E)** iron (Fe), **(F)** magnesium (Mg), **(G)** manganese (Mn), **(H)** phytic acid, **(I)** vitamin B1 (Vit B1), **(J)** vitamin B2 (Vit B2), **(K)** vitamin B6 (Vit B6), and **(L)** zinc (Zn). Associations were detected with 73,968 SNPs using the BLINK method for all traits except Fe and Mg; associations for Fe and Mg were detected with 318,644 SNPs using the MLMM method. Black solid line indicates Bonferroni threshold at 5% level, above which significant associations are depicted as red highlights.

Here, we report the results using genotypic parameters of MAF ≥ 0.02 (318 K SNPs) and MAF ≥ 0.02 + Ho ≤ 0.05 (74 K SNPs) for the multi-locus methods (Table 2), based on inflation values of Q-Q plots. The Manhattan plots show 20 significant MTAs detected for nine traits using BLINK with the 74 K matrix, explaining up to 12.18% PV (Figure 5), with five identified on both Ca1 and Ca4, followed by Ca5 (3), Ca6 (3), Ca2 (2), and Ca3 (2). A SNP locus (Ca3_31771545) associated with Zn content explained 12.18% PV, while another (Ca2_7953148) linked to Mn content explained 11.46% PV (Table 2). The MLMM with the 74 K matrix detected six significant MTAs for the same five traits, explaining 0.02–10.59% PV, with the MTAs for crude protein, Vit B6, and Zn validated across models. The 318 K SNP matrix detected 24 MTAs with BLINK, explaining up to 28.63% PV, and 12 MTAs with MLMM explaining 0.01–28.03% PV. Eight of these 36 MTAs were cross-validated across models for crude protein, Fo, and Vit B2 and B6. For instance, the MTA for Fo content (Ca5_664616) using BLINK explained 28.63% PV, while MLMM explained 28.03% PV. Importantly, both matrices and models validated a single association detected for crude protein on Ca6_57802709 (Supplementary Figure 2). Eight significant associations for Fo were detected using BLINK with the 318 K matrix, with two validated using the MLMM model (Supplementary Figure 3). One significant MTA for Fe was detected using MLMM with the 318 K matrix, while one MTA for Mg was detected on Ca4 using the MLMM model with both matrices. Most of the significant MTAs detected (Table 2) concentrated on Ca1, Ca3, Ca4, and Ca6.

**Table 2:** The MTAs detected for 12 nutritional traits using two multi-locus algorithms and two genotypic matrices based on two levels of Ho (≤ default in data and 0.05) with MAF ≥ 0.02.

S No	Marker ID	Linkage group	Position (bp)	*p* Value	FDR Adjusted *p* values	R^2^	Genotypic parameter	Matrix size	Method	Trait
1	Ca1_1,204,130	Ca1	1,204,130	3.46E-09	0.000	0.026	MAF_2	318,644	Blink	Zinc
2	Ca1_1,234,549	Ca1	1,234,549	0.000000114	0.036	0.008	MAF_2	318,644	MLMM	Iron
3	Ca1_6,576,624	Ca1	6,576,624	0.000000502	0.037	0.019	MAF_2_Ho_0.5	73,968	MLMM	Vitamin B2
4	Ca1_6,685,915	Ca1	6,685,915	1.37E-10	0.000	0.434	MAF_2	318,644	Blink	Vitamin B6
5	Ca1_7,031,080	Ca1	7,031,080	2.75E-12	0.000	0.000	MAF_2	318,644	Blink	Zinc
6	Ca1_13,432,853	Ca1	13,432,853	1.03E-08	0.000	0.112	MAF_2_Ho_0.5	73,968	Blink	Vitamin B1
7	Ca1_13,610,484	Ca1	13,610,484	1.84E-10	0.000	3.242	MAF_2_Ho_0.5	73,968	Blink	Manganese
8	Ca1_23,566,184	Ca1	23,566,184	6.66E-09	0.000	1.934	MAF_2_Ho_0.5	73,968	Blink	Vitamin B2
9	Ca1_32,272,158	Ca1	32,272,158	2.15E-09	0.000	0.002	MAF_2_Ho_0.5	73,968	Blink	Phytic acid
10	Ca1_32,272,158	Ca1	32,272,158	3.38E-10	0.000	0.002	MAF_2_Ho_0.5	73,968	Blink	Vitamin B1
11	Ca1_34,135,643	Ca1	34,135,643	1.02E-08	0.001	0.962	MAF_2	318,644	Blink	Vitamin B2
12	Ca2_7,953,148	Ca2	7,953,148	2.72E-09	0.000	11.455	MAF_2_Ho_0.5	73,968	Blink	Manganese
13	Ca2_30,146,046	Ca2	30,146,046	2.72E-08	0.001	9.314	MAF_2	318,644	Blink	Folate
14	Ca2_33,654,122	Ca2	33,654,122	6.14E-08	0.001	7.121	MAF_2_Ho_0.5	73,968	Blink	Vitamin B1
15	Ca2_34,025,270	Ca2	34,025,270	1.46E-08	0.002	10.977	MAF_2	318,644	MLMM	Vitamin B6
16	Ca3_3,519,666	Ca3	3,519,666	8.18E-13	0.000	25.651	MAF_2	318,644	Blink	Vitamin B2
17	Ca3_3,519,666	Ca3	3,519,666	5.88E-12	0.000	20.149	MAF_2	318,644	MLMM	Vitamin B2
18	Ca3_12,856,827	Ca3	12,856,827	4.86E-09	0.000	9.521	MAF_2_Ho_0.5	73,968	MLMM	Vitamin B6
19	Ca3_12,856,827	Ca3	12,856,827	8.35E-09	0.000	8.919	MAF_2	318,644	Blink	Vitamin B6
20	Ca3_31,771,545	Ca3	31,771,545	4.75E-11	0.000	12.180	MAF_2_Ho_0.5	73,968	Blink	Zinc
21	Ca3_31,771,545	Ca3	31,771,545	0.000000215	0.016	10.585	MAF_2_Ho_0.5	73,968	MLMM	Zinc
22	Ca3_31,771,545	Ca3	31,771,545	3.02E-08	0.002	11.023	MAF_2	318,644	Blink	Zinc
23	Ca3_37,989,135	Ca3	37,989,135	0.000000134	0.003	8.023	MAF_2_Ho_0.5	73,968	Blink	Vitamin B6
24	Ca3_37,989,135	Ca3	37,989,135	0.00000051	0.019	8.095	MAF_2_Ho_0.5	73,968	MLMM	Vitamin B6
25	Ca4_1,677,219	Ca4	1,677,219	6.83E-10	0.000	26.292	MAF_2	318,644	Blink	Folate
26	Ca4_4,224,251	Ca4	4,224,251	4.63E-08	0.001	6.181	MAF_2_Ho_0.5	73,968	Blink	Vitamin B1
27	Ca4_11,561,528	Ca4	11,561,528	0.000000166	0.003	0.675	MAF_2_Ho_0.5	73,968	Blink	Manganese
28	Ca4_13,749,741	Ca4	13,749,741	0.000000211	0.003	1.280	MAF_2_Ho_0.5	73,968	Blink	Vitamin B1
29	Ca4_14,274,204	Ca4	14,274,204	2.6E-09	0.000	3.516	MAF_2	318,644	Blink	Vitamin B6
30	Ca4_16,525,546	Ca4	16,525,546	6.65E-08	0.002	7.957	MAF_2_Ho_0.5	73,968	Blink	Vitamin B6
31	Ca4_17,620,596	Ca4	17,620,596	6.86E-10	0.000	6.297	MAF_2	318,644	Blink	Vitamin B6
32	Ca4_22,136,316	Ca4	22,136,316	4.15E-12	0.000	24.093	MAF_2	318,644	Blink	Vitamin B2
33	Ca4_22,136,316	Ca4	22,136,316	1.66E-11	0.000	22.984	MAF_2	318,644	MLMM	Vitamin B2
34	Ca4_25,983,206	Ca4	25,983,206	5.46E-08	0.017	8.554	MAF_2	318,644	MLMM	Magnesium
35	Ca4_29,622,277	Ca4	29,622,277	1.56E-09	0.000	4.554	MAF_2	318,644	Blink	Vitamin B6
36	Ca4_29,622,277	Ca4	29,622,277	1.48E-10	0.000	4.020	MAF_2	318,644	MLMM	Vitamin B6
37	Ca4_30,171,713	Ca4	30,171,713	2.73E-10	0.000	3.874	MAF_2	318,644	Blink	Vitamin B6
38	Ca4_37,846,900	Ca4	37,846,900	3.85E-09	0.000	2.335	MAF_2	318,644	Blink	Folate
39	Ca4_38,936,863	Ca4	38,936,863	0.000000673	0.050	4.577	MAF_2_Ho_0.5	73,968	MLMM	Magnesium
40	Ca4_40,475,284	Ca4	40,475,284	5.69E-09	0.000	2.419	MAF_2_Ho_0.5	73,968	Blink	β-Carotene
41	Ca4_42,574,243	Ca4	42,574,243	3.67E-20	0.000	8.488	MAF_2	318,644	Blink	Folate
42	Ca4_42,574,243	Ca4	42,574,243	3.2E-11	0.000	7.214	MAF_2	318,644	MLMM	Folate
43	Ca5_664,616	Ca5	664,616	1.82E-12	0.000	28.631	MAF_2	318,644	Blink	Folate
44	Ca5_664,616	Ca5	664,616	4.93E-13	0.000	28.025	MAF_2	318,644	MLMM	Folate
45	Ca5_4,272,745	Ca5	4,272,745	0.000000142	0.006	9.051	MAF_2	318,644	Blink	Folate
46	Ca5_13,877,854	Ca5	13,877,854	1.51E-11	0.000	1.438	MAF_2_Ho_0.5	73,968	Blink	β-Carotene
47	Ca5_21,506,706	Ca5	21,506,706	2.62E-09	0.000	0.000	MAF_2_Ho_0.5	73,968	Blink	Vitamin B6
48	Ca5_28,949,260	Ca5	28,949,260	0.000000291	0.022	1.593	MAF_2_Ho_0.5	73,968	Blink	Calcium
49	Ca6_3,377,773	Ca6	3,377,773	2.41E-09	0.000	3.588	MAF_2	318,644	Blink	Folate
50	Ca6_8,778,763	Ca6	8,778,763	0.000000227	0.003	4.549	MAF_2_Ho_0.5	73,968	Blink	Manganese
51	Ca6_13,906,696	Ca6	13,906,696	1.55E-08	0.001	5.912	MAF_2	318,644	Blink	Folate
52	Ca6_28,329,273	Ca6	28,329,273	6.93E-08	0.006	9.146	MAF_2	318,644	Blink	Vitamin B2
53	Ca6_28,329,273	Ca6	28,329,273	2.35E-09	0.000	9.785	MAF_2	318,644	MLMM	Vitamin B2
54	Ca6_30,884,344	Ca6	30,884,344	5.38E-11	0.000	11.014	MAF_2	318,644	Blink	Zinc
55	Ca6_30,884,344	Ca6	30,884,344	0.000000138	0.044	10.896	MAF_2	318,644	MLMM	Zinc
56	Ca6_53,593,815	Ca6	53,593,815	9.95E-11	0.000	4.994	MAF_2_Ho_0.5	73,968	Blink	Manganese
57	Ca6_57,141,662	Ca6	57,141,662	0.000000133	0.011	3.345	MAF_2	318,644	MLMM	Vitamin B2
58	Ca6_57,802,709	Ca6	57,802,709	0.000000516	0.038	2.410	MAF_2_Ho_0.5	73,968	MLMM	Crude protein
59	Ca6_57,802,709	Ca6	57,802,709	0.000000106	0.034	1.743	MAF_2	318,644	Blink	Crude protein
60	Ca6_57,802,709	Ca6	57,802,709	5.76E-08	0.018	2.398	MAF_2	318,644	MLMM	Crude protein
61	Ca6_57,802,709	Ca6	57,802,709	1.04E-08	0.001	2.093	MAF_2_Ho_0.5	73,968	Blink	Crude protein
62	Ca7_46,160,992	Ca7	46,160,992	7.8E-11	0.000	6.647	MAF_2	318,644	Blink	Vitamin B6

We assessed the favorable allelic combinations between nine significant marker loci for seven nutrition traits detected within the annotated genes and analyzed the effect of major and minor alleles on controlling favorable higher micronutrient concentrations. The effect of major allele “C” for Zn on Ca1_1204130 accounted for 77.9% of the reference set accessions, with a higher nutrient concentration (2.14–4.58 mg/100 g) than the effect of minor allele “G” in the locus, which accounted for 13.6% of accessions. Four associations for Vit B1 were detected within the reported genes, with major alleles accounting for a high concentration range (0.15–0.5 mg/100 g). For example, major allele “T” on Ca1_13432853 accounted for 87% of population accessions with a high concentration range, while minor allele “C” accounted for 2.13%; major allele “T” on locus Ca1_32272158 accounted for 54% of accessions, while minor allele “C” accounted for 31.9%; major allele “A” on locus Ca4_4224251 accounted for 81.2% of accessions, while minor allele “T” accounted for 6.7%; major allele “G” locus on Ca4_13749741 accounted for 41.7% of accessions, while minor allele “T” accounted for 41.3%. The association on Ca1_32272158 was co-localized for phytic acid and Vit B1, with major allele “T” (mentioned above for Vit B1) contributing to high phytic acid concentrations (9.61–19.38 mg/g). For the association between Ca3_3519666 and Vit B2, the major allele “G” accounted for 50.9% of accessions, while minor allele “A” accounted for 49.8%, but there was no pronounced effect contributing to higher Vit B2 concentration. Similarly, for Ca4_1677219 associated with Fo, the major allele “G” accounted for 50.9% of the accessions, while the minor allele “T” accounted for 49%. For the association between Ca4_17620596 and Vit B6, major allele “G” accounted for 87% of accessions contributing to high Vit B6 concentrations (0.3–1.4 mg/100 g), while minor allele “A” accounted for 3.6%. A single association detected for crude protein on Ca6_57802709 had major allele “T” accounting for 87% of accessions contributing to a high protein concentration (19.98–25.41/100 g) and minor allele “A” accounting for 4.4%.

Further, we scrutinized the candidate genes associated with these nine significant MTAs detected for seven nutrition traits (Table 3). A SNP locus (Ca4_1677219) associated with seed Fo content was present within the *Ca_07795* gene on Ca4 and explained 26.29% PV. A SNP locus (Ca3_3519666) linked to Vit B2 content was present within the *Ca_12279* gene on Ca3 and explained 25.65% PV. In the case of Vit B1, four significant MTAs were present within *Ca_03836, Ca_04599, Ca_14108*, and *Ca_26128* gene, explaining 6.18–0.002% PV. The association detected for phytic acid and Vit B1 were co-localized (Ca1_32272158) within the protein-coding sequence of *Ca_26128* gene coding for cytochrome P450 714A1-like. A SNP locus (Ca4_17620596) for Vit B6 was localized within the *Ca_05368* gene and explained 6.30% PV. Furthermore, a SNP locus for crude protein (Ca6_57802709) and Zn (Ca1_1204130) were present within *Ca_13661* and *Ca_00148* genes, explaining 2.41 and 0.03% PV, respectively.

**Table 3:** Candidate gene analysis for significant MTAs detected for 12 nutritional traits in the chickpea reference set.

Marker ID	P value	R^2^	Matrix size	Method	Trait	Reference allele	Alternate allele	Effect	Effect Impact	Functional class	Codon change	Amino acid change	Gene name	Transcript BioType	Gene coding	Transcript ID	Sequence name	Sequence description	GO Biological process	GO Cellular component	GO Molecular function
Ca4_1,677,219	6.83E-10	26.29	318,644	Blink	Folate	T	C	INTRON	MODIFIER				Ca_07795	protein_coding	CODING	Ca_07795	Ca_07795	guanine nucleotide-binding protein subunit gamma 2	G protein-coupled receptor signaling pathway		
Ca3_3,519,666	8.18E-13	25.65	318,644	Blink	Vitamin B2	T	A	INTRON	MODIFIER				Ca_12279	protein_coding	CODING	Ca_12279	Ca_12279	PREDICTED: uncharacterized protein LOC105851819			
Ca4_17,620,596	6.86E-10	6.30	318,644	Blink	Vitamin B6	A	G	INTRON	MODIFIER				Ca_05368	protein_coding	CODING	Ca_05368	Ca_05368	dihydroorotate dehydrogenase (quinone)	‘*de novo*’ pyrimidine nucleobase biosynthetic process,'*de novo*’ UMP biosynthetic process,oxidation–reduction process	mitochondrial inner membrane,plasma membrane	dihydroorotate dehydrogenase activity
Ca4_4,224,251	4.63E-08	6.18	73,968	Blink	Vitamin B1	A	T	INTRON	MODIFIER				Ca_03836	protein_coding	CODING	Ca_03836	Ca_03836	ubiquitin carboxyl-terminal hydrolase-like protein	ubiquitin-dependent protein catabolic process,protein deubiquitination		thiol-dependent ubiquitin-specific protease activity
Ca6_57,802,709	0.000000516	2.41	73,968	MLMM	Crude protein	T	A	INTRON	MODIFIER				Ca_13661	protein_coding	CODING	Ca_13661	Ca_13661	clast3, related protein	proteasome assembly	proteasome complex,nucleus,cytosol	
Ca4_13,749,741	0.000000211	1.28	73,968	Blink	Vitamin B1	T	G	SYNONYMOUS_CODING	LOW	SILENT	Agg/Cgg	R61	Ca_04599	protein_coding	CODING	Ca_04599	Ca_04599	probable serine/threonine-protein kinase WNK9	protein phosphorylation, intracellular signal transduction	cytoplasm	protein serine/threonine kinase activity, ATP binding
Ca1_13,432,853	1.03E-08	0.11	73,968	Blink	Vitamin B1	T	C	INTRON	MODIFIER				Ca_14108	protein_coding	CODING	Ca_14108	Ca_14108	vacuolar sorting-associated-like protein	intracellular protein transport, vesicle-mediated transport	intracellular	
Ca1_1,204,130	3.46E-09	0.03	318,644	Blink	Zinc	C	G	INTRON	MODIFIER				Ca_00148	protein_coding	CODING	Ca_00148	Ca_00148	(3S,6E)-nerolidol synthase 1-like			magnesium ion binding, terpene synthase activity, carboxy-lyase activity, and thiamine pyrophosphate binding
Ca1_32,272,158	2.15E-09	0.00	73,968	Blink	Phytic acid	C	T	SYNONYMOUS_CODING	LOW	SILENT	cgC/cgT	R177	Ca_26128	protein_coding	CODING	Ca_26128	Ca_26128	cytochrome P450 714A1-like	oxidation–reduction process	integral component of membrane	monooxygenase activity, iron ion binding, oxidoreductase activity, and acting on paired donors, with incorporation or reduction in molecular oxygen, heme binding
Ca1_32,272,158	3.38E-10	0.00	73,968	Blink	Vitamin B1	C	T	SYNONYMOUS_CODING	LOW	SILENT	cgC/cgT	R177	Ca_26128	protein_coding	CODING	Ca_26128	Ca_26128	cytochrome P450 714A1-like	oxidation–reduction process	integral component of membrane	monooxygenase activity, iron ion binding, and oxidoreductase activity, acting on paired donors, with incorporation or reduction in molecular oxygen, heme binding

### Promising accessions with high nutrient content for chickpea improvement

Promising chickpea accessions with high nutrient content can be used as donors in breeding programs to develop improved varieties for meeting worldwide nutritional demand. Using the first approach, we identified 16 accessions from the reference set (referred to as G2, Supplementary Table 3) with trait values higher than the respective population median values for only SY, in different environments (Thudi et al., 2014).

**Figure 6:**
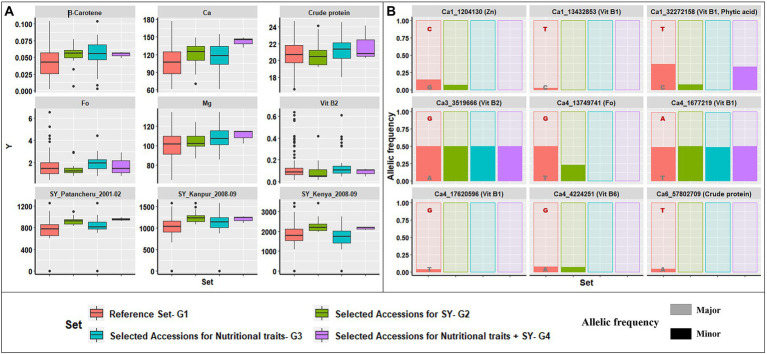
Selection strategy for identifying potential donor accessions. **(A)** Graphical representation of variability in trait content for four groups: G1, reference set; G2, selected accessions for high SY across five seasons and four locations; G3, selected accessions for high nutritional trait range; G4, common accessions from G2 and G3. Comparison of trait range for β-Carotene, Ca, crude protein, Fo, Mg, Vit B2, and SY between three locations, highlighting that G4 accessions had a narrow range and comparable or higher mean than G1. **(B)** Variation in the major and minor allelic frequencies for the nine loci detected significance for six traits within the reported genes for G1–G4. The major allele was dominant for selected accessions in G2, G3, and G4, compared to the reference set, G1.

Using data for 12 nutritional traits, a similar selection for superior trait range revealed 33 accessions (referred to as G3) with desirable trait values higher than the respective population median values. The selected accessions (Supplementary Table 4) were narrowed to three accessions (referred to as G4), by comparing G2 and G3, to identify accessions with superior performance for both the 12 nutritional traits and agronomic traits from different environments (Supplementary Table 5). An overview of the superior performance of G2, G3, and G4 over G1 is presented in Figure 6. Figure 6A includes six nutrition traits (β-Carotene, Ca, crude protein, Fo, Mg, and Vit B2) and SY recorded at three locations under rainfed ecology (Patancheru, Kanpur, and Egerton University, Kenya). The 33 accessions in G3 selected for superior nutritional traits had higher mean performance and narrower range than G1 for the six nutrition traits depicted.

Similarly, the G2 accessions had higher mean performance and a narrower range than G1 for various agronomic and physiological traits. However, G2 accessions had a lower mean performance for nutritional traits than G3 accessions. Thus, we identified three accessions with superior performance for agronomic and nutritional traits (G4). Supplementary Figure 4 shows the correlation between nutrition and agronomic traits, with negative correlations for some traits (crude protein, Fe, Mn, and Zn). The G4 accessions will be a major advantage for incorporating into breeding programs targeting abiotic stress tolerance and biofortification.

The contribution of major alleles identified in G1–G4 for the nine significant MTAs detected within reported genes is depicted in Figure 6B. The major alleles at each locus in all four groups made varying yet significant contributions to the favorable higher trait range. Of the nine loci analyzed, only one (Ca1_32272158) had a lower major allele contribution in G4 than G3, while the remainder had similar contributions, validating the premise of selecting superior accessions for nutrition and agronomic traits.

In the second approach to identify potential donors, we analyzed the correlations between the 12 nutritional traits, and SY and 100SW from different environments, as described in Thudi et al. (2014). The PCA factor graph showed that PC1 and PC2 accounted for 21.5 and 16.6% of the variation in the measured traits, respectively (Supplementary Figure 5). Here, SY and 100SW were closely related to Vit B1 and B6 and distantly related to crude protein and phytic acid content. β-Carotene content was closely associated with phytic acid levels in chickpea seeds. Moreover, Fo was closely related to Zn, Fe, and Mg levels. Hierarchical cluster analysis classified the 14 traits into three distinct clusters: (1) 100SW, crude protein, and phytic acid; (2) Mn, Fo, Zn, Fe, β-Carotene, SY, and Vit B1, B2, and B6; and (3) Mg and Ca (Supplementary Figure 6). The top 30 accessions (1% of reference set) were selected based on phenotypic performance for these 14 traits. Accessions grouped in the same cluster were compared to identify accessions for favorable combinations of two or more traits. For instance, cluster 1 had two accessions with higher 100SW, crude protein and lower phytic acid content, four accessions with higher 100SW and crude protein, four accessions had higher crude protein and lower phytic acid, and three accessions with higher 100SW and minimal phytic acid content (Supplementary Table 6). Cluster 2 had one accession had higher Fe, Zn, Mn, and Vit B1 and B6, one accession had higher Fe, Vit B1, Fo, and Mn, one accession had higher Fe, Zn, and Vit B1 and B2, one accession had higher Fe, Fo, and Vit B1 and B2, one accession had higher Zn, Mn, and Vit B1 and B6, one accession had higher β-Carotene, Fo, and B1 and B6, and one accession had higher Zn, Fo, Mn, and Vit B1 (Supplementary Table 7). In cluster 3, eight accessions had higher Ca and Mg content (Supplementary Table 8).

Selected intercrossing may facilitate the development of improved accessions harboring beneficial alleles for nutritional and yield traits in chickpea. For example, ICC15406 and ICC13124 (cluster 1) could be crossed with ICC6279, ICC3582, ICC2720, ICC8752, ICC16915, ICC11584, ICC7413, or ICC2679 (cluster 3) to breed large-seeded chickpea varieties with high Ca, Mg, and crude protein, and low phytic acid content. ICC6875 (cluster 2) could be crossed with ICC15406 or ICC13124 (cluster 1) to breed high Fe, Zn, Mn, Vit B1 and B6, 100SW, crude protein, and low phytic acid varieties. ICC10755, ICC7272, ICC6306, and ICC8350 (cluster 1) could be crossed with the high-yielding accession (ICC12037). ICC13816 could be crossed with high-yielding accessions (ICC1164 and ICC13764) to breed high-yielding large-seeded varieties with enhanced crude protein and Vit B1 and B6. We also compared the accessions identified in both approaches to reveal common accessions—seven accessions (ICC10399, ICC1392, ICC1710, ICC2263, ICC1431, ICC4182, and ICC16915)—that could be used in breeding programs as potential donors to enhance both, chickpea micronutrient content and productivity.

## Discussion

Micronutrient malnutrition is characterized by a chronic lack of vitamins and minerals in the human diet. For instance, a lack of micronutrients, such as Fe, Fo, β-Carotene, and Vit B12 can cause anemia. An estimated 42% of children under 5 years of age and 40% of pregnant women suffer from anemia globally (World Bank Data, 2016). Notably, iron-folic acid supplementation is crucial for pregnant women to avoid maternal anemia, puerperal sepsis, low birth weight, and preterm birth (WHO, 2016). Therefore, enriching chickpea seeds with such micronutrients would make it a complete dietary source to address micronutrient malnutrition in developing countries. A broad range of variation in the 12 nutritional traits was observed in the chickpea reference set in the present study. Furthermore, we report SNPs associated with 12 nutritional traits and potential donors that can be deployed in breeding programs to develop biofortified chickpeas.

Understanding the genetic basis of interactions between micronutrients, such as the synergistic effect of Fe, crude protein, and the vitamin complex or the competitive effect of β–Carotene and phytic acid with the vitamin complex and bioconversion factors, is crucial for developing nutrient-rich crops. Nutrient bioavailability depends on endogenous (phytic acid, fiber, amino acids, and proteins) and exogenous factors in seeds. Legumes contain some promoters that enhance mineral bioavailability, even in the presence of anti-nutrients. Promoter compounds are natural plant metabolites, and only minor changes in their accumulation in seeds may be necessary to impact the bioavailability of micronutrients such as inulin, found in small quantities in raw samples of lentil, chickpea, red kidney bean, common white bean, white bean, and faba bean (Rastall and Gibson, 2015). The present study provided important insights into the relationships among different nutrition traits in chickpeas.

Population stratification has been established in the chickpea reference set (Thudi et al., 2021), with three clusters/subpopulations independent of biological status and seed type. In the present study, we detected three subpopulations with ADMIXTURE in the reference set, including 258 accessions. In accordance with the results obtained in this study, a recent study by Varshney et al. (2019) also reported the presence of three subpopulations using genome-wide SNP markers. In another study, four subpopulations were revealed in a diverse set consisting of 186 chickpea genotypes, using DArT-seq markers (Farahani et al., 2019).

Integrating genome-wide sequence information with precise phenotypic variation has the potential to detect accessions with casual variants that may be responsible for essential phenotypes such as enhanced micronutrient concentration. GWAS overcomes two common limitations of the traditional linkage mapping (*viz.* restricted allelic diversity and limited genetic resolution; Brachi et al., 2011; Huang and Han, 2014). Owing to its high resolution, cost effectiveness and non-essential pedigrees, association mapping has been able to dissect many important traits in chickpea, such as concentration of mineral nutrients (Diapari et al., 2014; Upadhyaya et al., 2016; Fayaz et al., 2022; Samineni et al., 2022); seed yield (Basu et al., 2018); drought tolerance (Li et al., 2018); root morphology, phosphorous acquisition, and use efficiency (Thudi et al., 2021); and salinity tolerance (Ahmed et al., 2021).

The major challenge for GWAS is to control the false positives, primarily caused by population structure and family relatedness (Kaler et al., 2020). While the single-locus methods (like mixed linear model, MLM) address this challenge by incorporating the two confounding factors as covariates (Price et al., 2006), overfitting in the model frequently results in false-negatives that might exclude key loci. In this regard, the multi-locus models are a better alternative to overcome the false-negatives (Zhang et al., 2019). Among the multi-locus GWAS methodologies, MLMM uses marker-trait association tests to select associated markers that are fitted as cofactors. These cofactors are then adjusted in the mixed model by forward and backward stepwise regression (Segura et al., 2012). Another multi-locus method, BLINK, developed recently has demonstrated improved statistical power compared to other multi-locus methods. BLINK removes the assumption that causal variants be evenly distributed across the genome, as required by the SUPER (settlement of MLM under progressively exclusive relationship) and FarmCPU (fixed and random model circulating probability unification) methods, making the model superior in statistical power with discovery of less false positives. In addition, BLINK reduces the computing time remarkably (Huang et al., 2019). Taking this into consideration, three statistical algorithms—one single-locus (MLM) and two multi-locus (MLMM and BLINK)—were utilized in the present study to detect genome-wide association signals for 12 nutritional traits. As reflected in Table 2, BLINK method was superior out of the three statistical algorithms used the present study, detecting 44 of the 62 MTAs reported using 318 and 74 K matrices. Out of these 44 MTAs, one (Ca1_32272158) co-localized for Vit B1 and phytic acid; eight (Ca3_3519666, Ca4_22136316, Ca4_29622277, Ca4_42574243, Ca5_664616, Ca6_28329273, Ca6_30884344, and Ca6_57802709 for Vit B2, Vit B2, Vit B6, Fo, Fo, Vit B2, Zn, and crude protein, respectively) were validated by both MLMM and BLINK algorithms with 318 K matrix; and three (Ca3_31771545, Ca3_37989135, and Ca6_57802709 for Zn, Vit B6, and crude protein, respectively) were validated by both MLMM and BLINK algorithms for 74 K matrix. To ascertain the validity of our results, the MTAs for various nutritional traits identified in the present study were compared with some previous association mapping studies in chickpea. Under control conditions, Samineni et al. (2022) identified MTAs for Fe on Ca4; for Zn on Ca1, Ca4, and Ca7; while majority of the MTAs for protein content were identified on Ca1, Ca4, and Ca6. Furthermore, seven MTAs for seed protein content were mapped on five kabuli chromosomes including Ca1, Ca2, Ca4, Ca6, and Ca7 (Upadhyaya et al., 2016). In accordance with these studies, the present study identified a key MTA for crude protein content on Ca6 using BLINK and MLMM algorithms, for both 74 and 318 K matrices. Therefore, the tightly linked marker for the MTA on Ca6 (Ca6_57802709) holds promise for further validation using diverse populations and could be deployed for early generation selections in breeding programs. The MTAs identified for remaining 11 nutritional traits in the present study have not been reported previously and seem to represent novel genetic loci controlling grain nutritional content in chickpea.

A total of nine significant MTAs for seven nutritional traits were associated with putative genes. For instance, one MTA detected for Fo on Ca1_1677219 was within the *Ca_07795* gene, coding for guanine nucleotide-binding protein subunit gamma 2, with a molecular role in the G protein-coupled receptor signaling pathway. An association was identified between crude protein and the genomic region coding for a clast3-related protein responsible for proteasome assembly in the nucleus and cytosol. The association detected for phytic acid (Ca1_32272158) co-localized with the association for Vit B1 within the protein-coding sequence of cytochrome P450 714A1 gene (*Ca_26128*) involved in the oxidation–reduction process. This protein is an integral component of the cellular membrane and is responsible for regulating monooxygenase activity, iron ion binding, and oxidoreductase activity, acting on paired donors, incorporating or reducing molecular oxygen and heme-binding (Zhang et al., 2011). For Vit B1, three more associations were detected within *Ca_14108*, *Ca_03836*, and *Ca_04599*. The gene *Ca_14108* codes for an intracellular vacuolar sorting-associated-like protein, *Ca_03836* is responsible for coding ubiquitin carboxyl-terminal hydrolase-like protein and is involved in protein deubiquitination, and *Ca_04599* plays a role in protein phosphorylation and intracellular signal transduction *via* serine/threonine-protein kinase WNK9. For Vit B2, the MTA (Ca3_3519666) is present within *Ca_12279*, predicted to code for an uncharacterized protein. The MTA (Ca4_17620596) identified for Vit B6 lies within the *Ca_05368* gene, responsible for producing dihydroorotate dehydrogenase (quinone) in mitochondrial inner membrane and plasma membrane for dihydroorotate dehydrogenase activity (Ullrich et al., 2002). The MTA (Ca1_1204130), associated with Zn and detected within the *Ca_00148* gene, codes for the (3S,6E)-nerolidol synthase 1-like protein involved in magnesium ion binding, terpene synthase activity, carboxylase activity, and thiamine synthesis (Degenhardt and Gershenzon, 2000). The incorporation of identified genes to develop nutrient-rich legume varieties through genetic engineering or molecular breeding in an integrated approach will provide effective and long-term solutions to the increasing problem of malnutrition.

Integrating genomic resources with breeding efforts by exploiting various diversity panels to develop superior, biofortified, and climate-resilient varieties is imperative for addressing the emerging constraints limiting chickpea production and micronutrient malnutrition (Roorkiwal et al., 2020; Varshney et al., 2021a,b,c). Based on nutritional and yield-related traits, the promising accessions identified in this study can serve as potential donors for designing nutrient-rich chickpea varieties for the future.

## Data availability statement

The original contributions presented in the study are included in the article/Supplementary material; further inquiries can be directed to the corresponding authors.

## Author contributions

RKV and MR conceived and designed the experiments. MR, SP, AB, and RB performed the experiments. AB, RB, PB, and VV analyzed the data. MR, AC, CB, and RKV contributed to the reagents, materials, and analysis tools. MR, AB, and RB wrote the first draft of the manuscript, and KHMS edited and revised the manuscript. All authors contributed to the article and approved the submitted version.

## Funding

RKV is grateful to Bill & Melinda Gates Foundation, United States (Grant No OPP1114827), and Department of Biotechnology, Ministry of Science & Technology, Government of India for supporting this research in parts. The authors thank due to the Science & Engineering Research Board (SERB) of Department of Science & Technology (DST), Government of India for providing the J C Bose National Fellowship (SB/S9/Z-13/2019) to RKV, MR, and SP, and the Department of Science and Technology, Government of India for providing funding support through the INSPIRE Faculty Scheme, Early Career Research Award—SERB and SERB-NPDF (PDF/2016/003379), respectively.

## Conflict of interest

The authors declare that the research was conducted in the absence of any commercial or financial relationships that could be construed as a potential conflict of interest.

## Publisher’s note

All claims expressed in this article are solely those of the authors and do not necessarily represent those of their affiliated organizations, or those of the publisher, the editors and the reviewers. Any product that may be evaluated in this article, or claim that may be made by its manufacturer, is not guaranteed or endorsed by the publisher.

## Footnotes

^1^ https://fdc.nal.usda.gov/fdc-app.html#/food-details/173756/nutrients
