# Supplementary material for: Genome-wide association mapping of nutritional traits for designing superior chickpea varieties
Source: Front Plant Sci. 2022 Aug 23;13:843911. doi: 10.3389/fpls.2022.843911 (PMC9445663; doi:10.3389/fpls.2022.843911)
Supplement: Supplementary file 1 [file Data_Sheet_1.docx]

**Genome-wide association mapping of nutritional traits for designing superior chickpea varieties**

Manish Roorkiwal^1,2,3,*^, Aditi Bhandari^1^, Rutwik Barmukh^1^, Prasad Bajaj^1^, Vinod Kumar Valluri^1^, Annapurna Chitikineni^1^, Sarita Pandey^1^, Bharadwaj Chellapilla^2,4^, Kadambot H.M. Siddique^2^, Rajeev K Varshney^1,2,5*^

^1^Center of Excellence in Genomics & Systems Biology, International Crops Research Institute for the Semi-Arid Tropics (ICRISAT), Hyderabad-502324, India

^2^The UWA Institute of Agriculture, The University of Western Australia, Perth 6001, Western Australia, Australia

^3^Khalifa Center for Genetic Engineering and Biotechnology (KCGEB), United Arab Emirates University, Al Ain, Abu Dhabi, UAE

^4^ICAR- Indian Agricultural Research Institute (IARI), New Delhi-110012, India

^5^State Agricultural Biotechnology Centre, Centre for Crop and Food Innovation, Murdoch University, Murdoch, Western Australia, Australia

*** Correspondence: mroorkiwal@uaeu.ac.ae (Manish Roorkiwal);

[rajeev.varshney@murdoch.edu.au](mailto:rajeev.varshney@murdoch.edu.au) (Rajeev K. Varshney)

**Supplementary Figures**

**
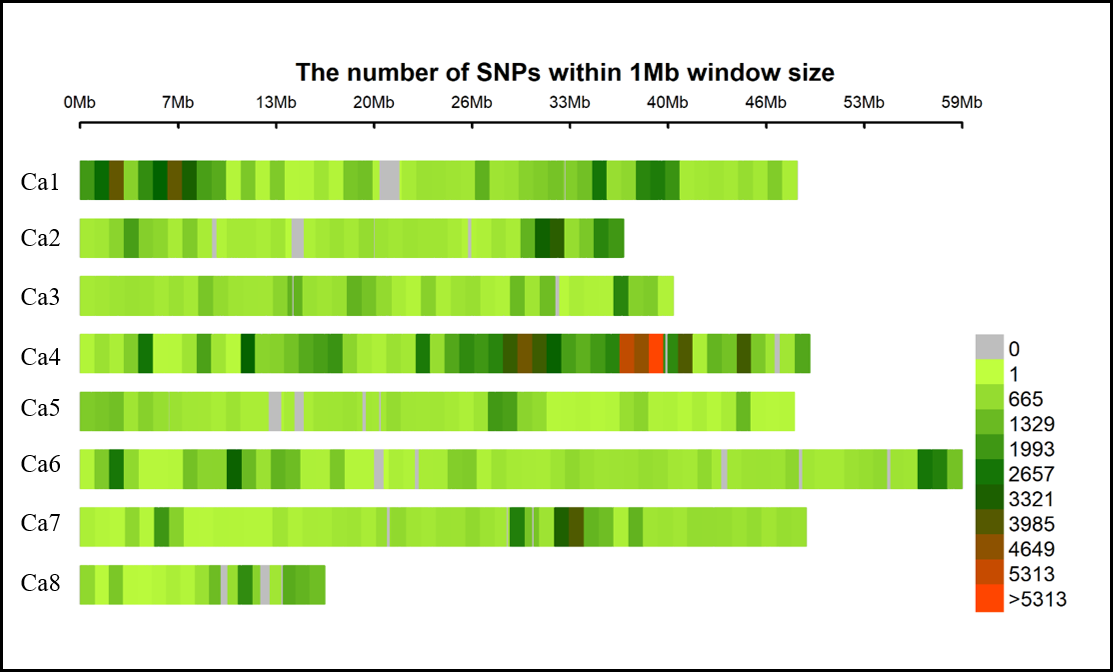
**

**Supplementary Figure 1:** **Heatmap showing uneven distribution of 318,644 SNPs along the eight pseudomolecules.**

**
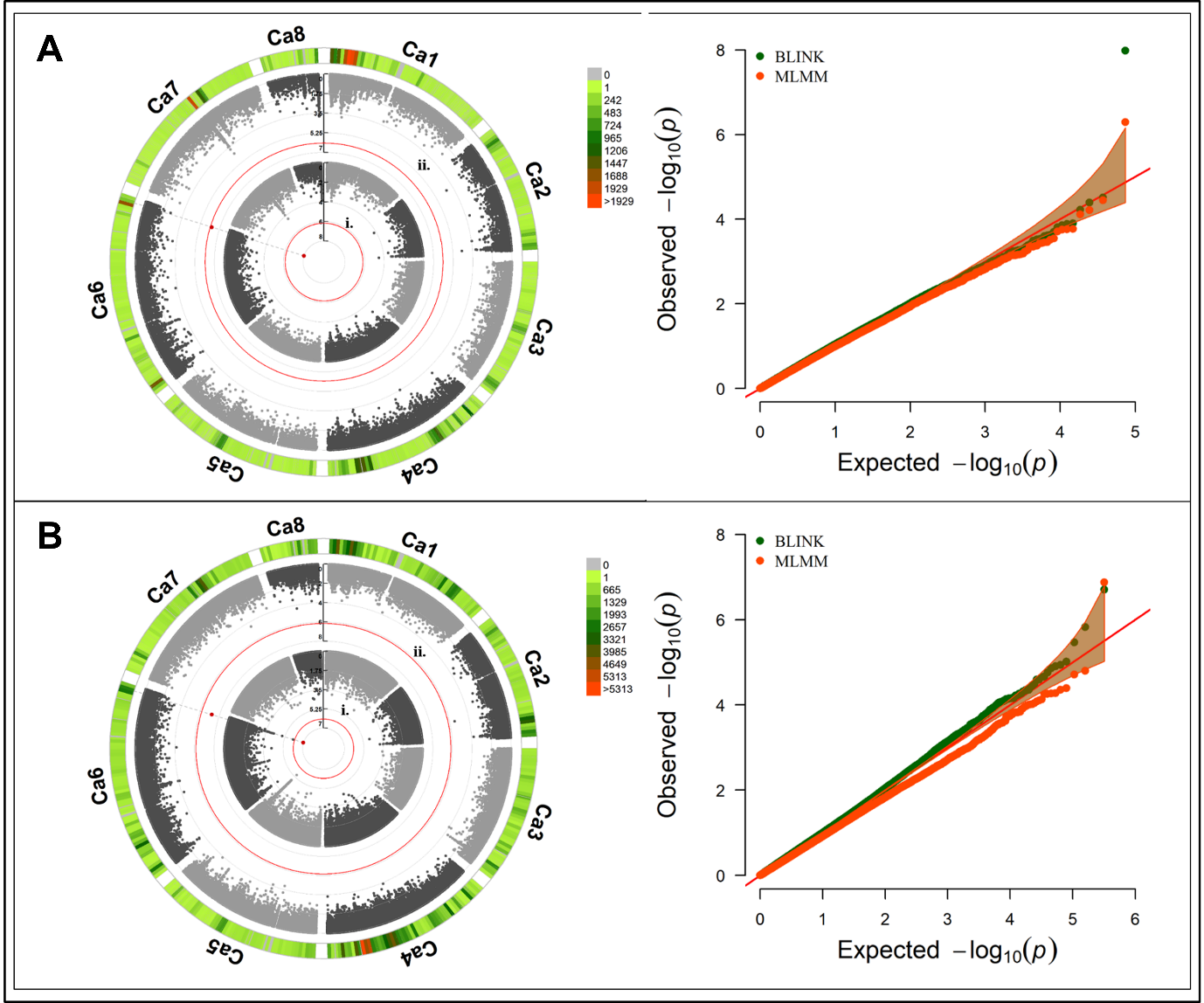
**

**Supplementary Figure 2:** **Manhattan plots and Q-Q plots showing associations between crude protein and different SNP matrices and algorithms.** GWAS signal for crude protein obtained with **(**A) 73,968 SNPs and (B) 318,644 SNPs in the reference set using (i) BLINK and (ii) MLMM methods. Red dashed line indicates the Bonferroni threshold at the 5% level. Significant associations were validated across the two matrices and two methods, as depicted in the circular Manhattan plots. Both methods were effective and comparable in controlling the detection of false positives, as depicted in the Q-Q plots.

**
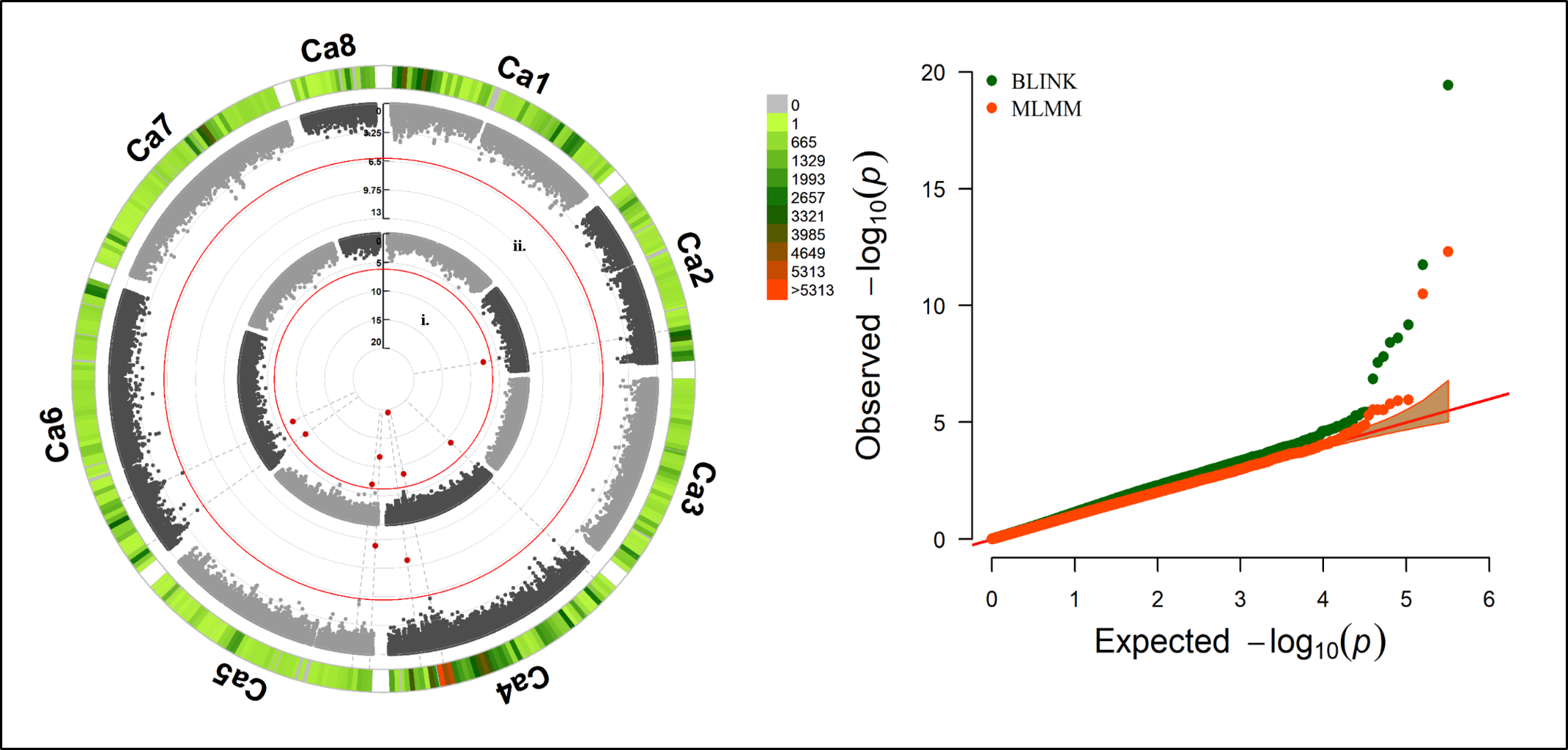
**

**Supplementary Figure 3:** **Manhattan plot and Q-Q plot showing association for folate content in chickpea seed.** GWAS signal for folate content obtained with 318,644 SNPs in the reference set using (i) BLINK and (ii) MLMM methods. Red dashed line indicates the Bonferroni threshold at the 5% level. Of the eight significant associations detected for folate, two were validated across the two methods, as depicted in the circular Manhattan plot. Both methods were effective and comparable in controlling the detection of false positives, as depicted in the Q-Q plot.

**
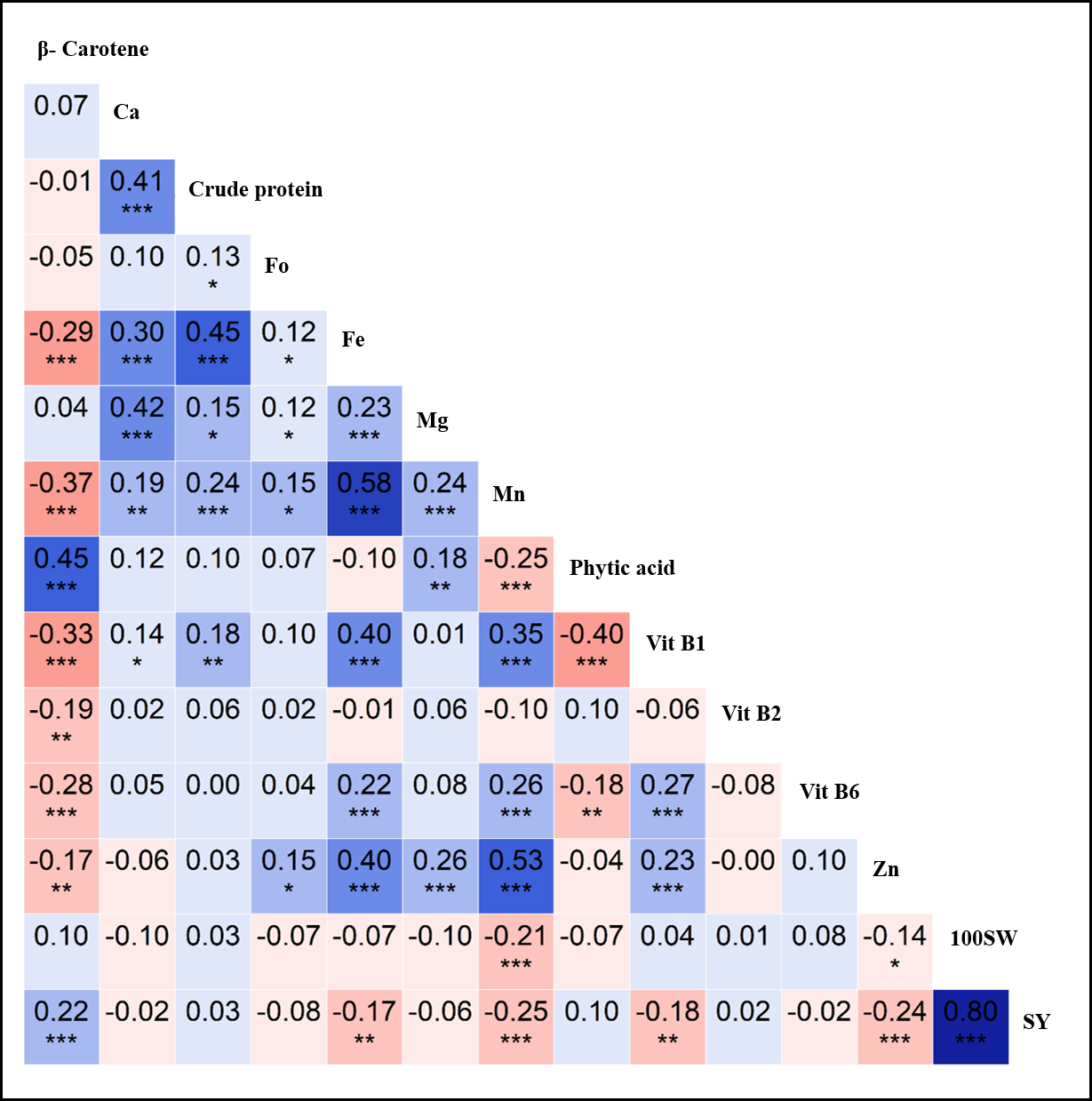
**

**Supplementary Figure 4:** **Correlation analysis of 12 nutritional traits and two agronomic traits using the chickpea reference set.** Pearson’s r-values showing correlations between 12 traits [β-Carotene, calcium (Ca), crude protein, folate (Fo), iron (Fe), magnesium (Mg), manganese (Mn), phytic acid, vitamin B1 (Vit B1), vitamin B2 (Vit B2), vitamin B6 (Vit B6), zinc (Zn)] and two agronomic traits [100-seed weight (100SW) and seed yield (SY)], for the reference set. Blue indicates positive correlations, and red indicates negative correlations among traits; color intensity depicts correlation strength. *significant at < 0.05 level, **significant at <0.01 level, *** significant at < 0.001 level, blank for non-significant.

**
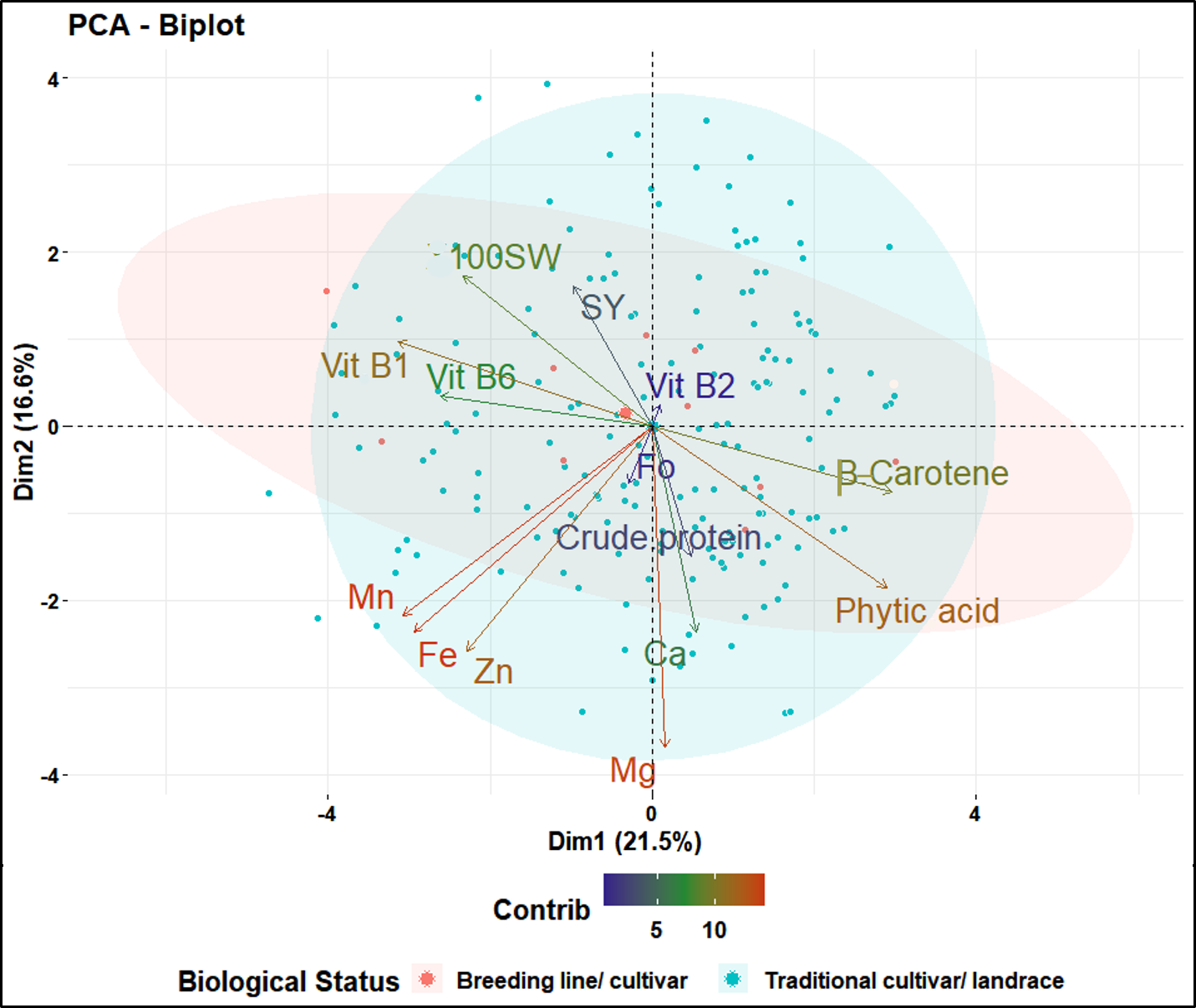
**

**Supplementary Figure 5:** **Principal component analysis for 12 nutritional traits and two agronomic traits.** Projection of 258 accessions of the reference set on the first plane of principal component analysis using phenotypic data for 12 nutritional traits [β-Carotene, calcium (Ca), crude protein, folate (Fo), iron (Fe), magnesium (Mg), manganese (Mn), phytic acid, vitamin B1 (Vit B1), vitamin B2 (Vit B2), vitamin B6 (Vit B6), and zinc (Zn)] and two agronomic traits [100-seed weight (100SW) and seed yield (SY)]. The first two components, PC1 and PC2, explain 32.1% of the variance between genotypes. Among the traits, Mg and Mn account for the highest variances, while crude protein, Fo, and Vit B2 account for the least. There is no evident clustering based on the biological status of the accessions.

**
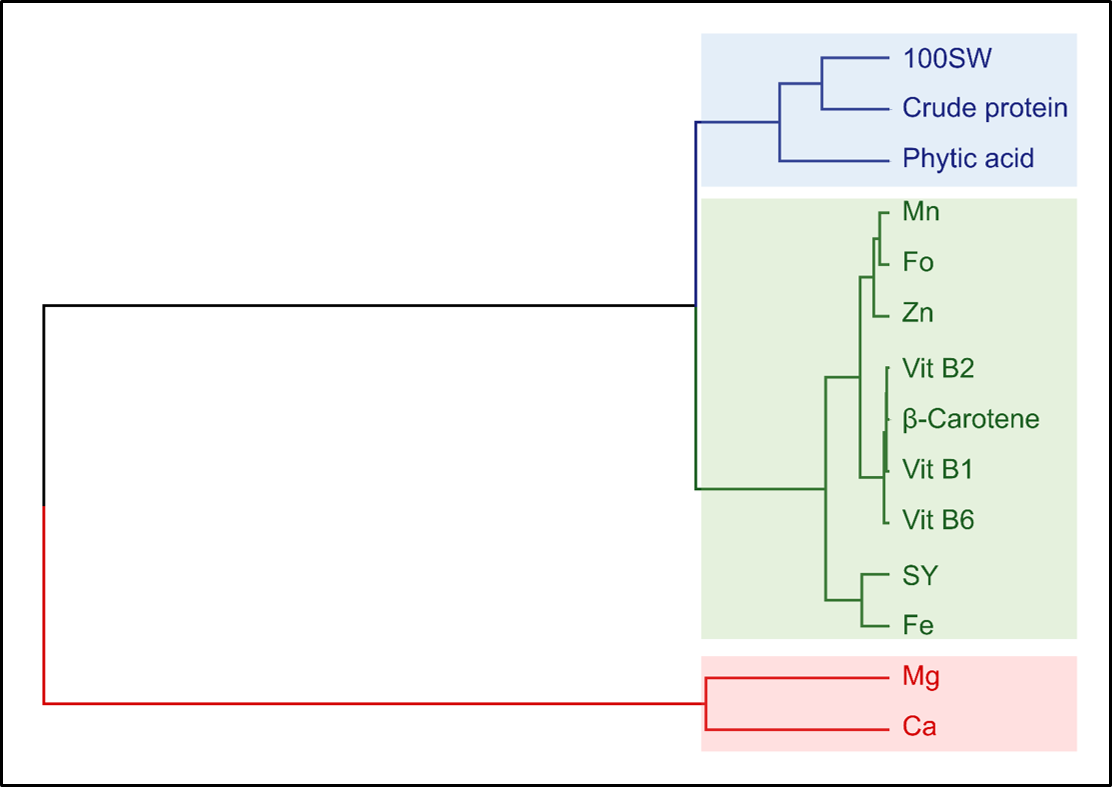
**

**Supplementary Figure 6: Hierarchical cluster analysis for 12 nutritional and two agronomic traits**. Twelve nutritional traits [β-Carotene, calcium (Ca), crude protein, folate (Fo), iron (Fe), magnesium (Mg), manganese (Mn), phytic acid, vitamin B1 (Vit B1), vitamin B2 (Vit B2), vitamin B6 (Vit B6), zinc (Zn)] and two agronomic traits [100-seed weight (100SW) and seed yield (SY)] were grouped into three distinct clusters: (1) 100SW, crude protein, and phytic acid; (2) Mn, Fo, Zn, Fe, β-Carotene, Vit B1, Vit B2, Vit B6, and SY; (3) Mg and Ca.
